# The Impact of Living With Inducible Laryngeal Obstruction

**DOI:** 10.1111/cea.70026

**Published:** 2025-03-05

**Authors:** Siobhan Ludlow, Lucie Byrne‐Davis, Stephen J. Fowler

**Affiliations:** ^1^ Medical Education, School of Medical Sciences University of Manchester Manchester UK; ^2^ NIHR Manchester Biomedical Research Centre Manchester University NHS Foundation Trust Manchester UK; ^3^ Division of Infection, Immunity and Respiratory Medicine, School of Biological Sciences University of Manchester Manchester UK

1

**Summary**

- Inducible laryngeal obstruction can be life‐changing and significant adaptations are often needed.
- Individualised treatment plans for inducible laryngeal obstruction are required that address patients' priorities.

To the Editor,

1

Inducible laryngeal obstruction (ILO) is a debilitating condition which typically presents with a sudden onset of breathlessness due to transient upper airway obstruction [1]. The prevalence of ILO is unknown but is more common in females with a broad age range [2, 3].

Episodes of ILO are typically sudden, unpredictable and vary in severity [2]. They are elicited by everyday things such as strong scents [4, 5], and key symptoms of ILO include dyspnoea, coughing and throat tightness [6]. Individuals present across a variety of healthcare settings [2] and attract unnecessary medical treatment and increased healthcare utilisation [7].

To our knowledge, our study is the first to describe the impact of living with ILO, focussing on the burden of disease on all aspects of life. Other studies have not considered the impact of ILO on the impairment, functioning and disability in its entirety [8].

We aimed to explore the lived experiences of patients with ILO and their perceptions of the disease. The study participants were adults (≥ 17 years) recruited from the regional complex breathlessness service based at Manchester University NHS Trust, UK. Participants were eligible if they had a diagnosis of ILO confirmed on laryngoscopy.

In 2024, SL conducted 15 semi‐structured interviews (40–100 min). The interview guide was developed considering previous literature and advice from our patient and public involvement (PPI) group, comprising patients living with ILO. The guide focused on (1) the physical, social and psychological impact of living with ILO; (2) how ILO made patients feel; (3) the public and professional understanding of ILO; (4) management of ILO; and (5) priorities for the future.

Data analysis (Figure 1) revealed five major themes: illness burden; loss of autonomous life; anxiety and fear for the future; social isolation and loneliness; coping strategies, support and management. Participants discussed feeling their symptoms were often not understood by healthcare professionals and they were not believed (‘Just being passed around different professionals, I just didn't know whether I was being listened to […] and whether they didn't know what to do’. ID05, female, aged 53). The illness was perceived as a burden and participants were constantly reminded of the sudden, unpredictable nature of ILO and its chronic status (‘That's the disappointment for me that I can't be cured. It's something I've got to live with […]’, ID04, female, aged 49). Participants discussed one of the biggest impacts being the loss of autonomy and freedom. Many discussed the fear of going out alone and relying on family members to always be with them (‘I never go out on my own […], I've always got somebody with me’, ID015, female, aged 59). When attending social events, many spoke about avoiding environments to keep control of their symptoms. One participant discussed when attending barbeques at friends' houses, they had to stay inside whilst the food was being cooked (‘[…] you feel like you're being left out. At a BBQ, that first part is all about standing around, taking in all the smells, seeing people cooking on food. You don't get that anymore’, ID017, male, aged 58). For some, ILO affected the feasibility of planning for the future (‘[…] If we were to plan a day to go out or had something booked. The fear of having to cancel that […]’, ID05, female, aged 53). Living with ILO caused participants to anticipate or predict when attacks may occur, (‘[…] the mind battles against whether you should go out and allow it to be triggered or whether you should hold back on stuff’, ID05, female, aged 53). Participants discussed setting realistic goals and learning to live with ILO (‘[…] when something goes on for so long, you just get used to it and you adjust to certain things’, ID05, female, aged 53). Participants discussed being honest about their ILO diagnosis and it now being part of them (‘[…] I own it. It is part of me. I'm better off being open about it and then people hopefully will be OK’, ID011, female, aged 52).

**FIGURE 1:**
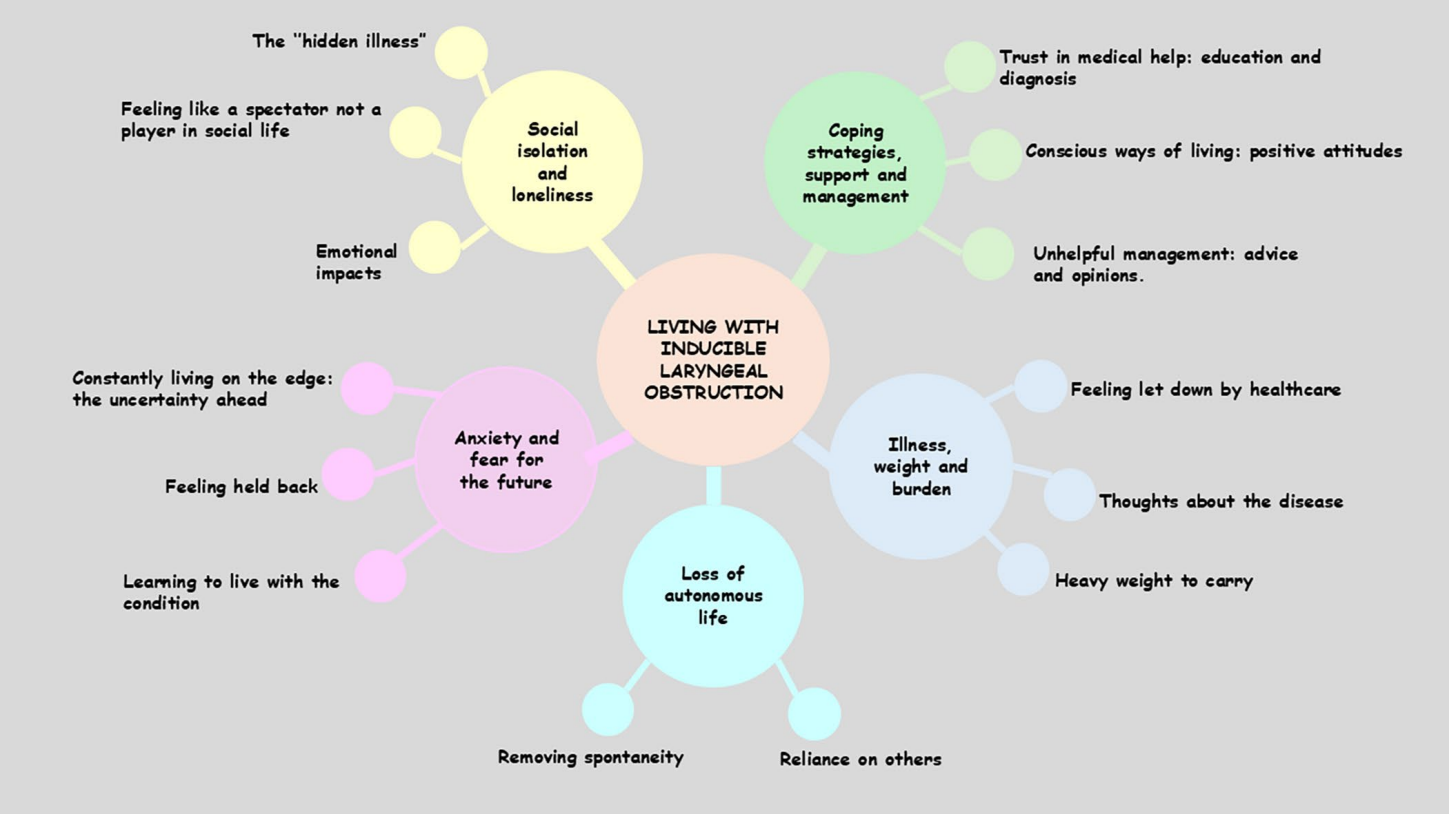
Visual representation of themes and subthemes.

Our results are consistent with the literature showing ILO has often been misinterpreted by patients and clinicians for some time prior to diagnosis [6]. Participants discussed the importance of receiving a definitive diagnosis of ILO and the feelings of relief this gave. Several factors contributed to the psychosocial burden of ILO including: (1) frustration at the long waits for diagnosis and not wanting to attend appointments for fear of not being believed or understood; (2) the variable and unpredictable nature of ILO; (3) the impact ILO had on the ability to socialise and work. The diagnosis is not easy to make [6], and the need for multidisciplinary assessment is required for a more personalised and patient‐centred care [9].

The data highlight the huge psychosocial burden ILO has on individuals, and identification of these challenges and psychosocial factors is needed to support education and training. We hope that the findings will be used to increase health care professionals' awareness of living with ILO from a patient perspective.

Our cohort of patients was all adults, and it would be interesting to see the impact of ILO on younger people. Patients were recruited from a tertiary referral centre and therefore may be indicative of more severe ILO. Additional information about the methods, findings and discussion is available in the following repository: https://doi.org/10.5281/zenodo.14501352. Future work is required to provide individualised patient‐centred treatment plans and consider the development of outcome measures that capture the broad impact highlighted by our study.

## Author Contributions

**Siobhan Ludlow:** conceptions and design of study, data collection, data analysis and interpretation, drafting of article and writing. **Lucie Byrne‐Davis:** conceptions and design of study and review and editing. **Stephen J. Fowler:** conceptions and design of study and review and editing.

## Disclosure

Transparency declaration: All authors affirm that the manuscript is an honest, accurate and transparent account of the study being reported. No important aspects of the study have been omitted.

## Ethics Statement

The study was approved by Greater Manchester West Research Ethics Committee (23/NW/0198), and all participants gave written informed consent.

## Conflicts of Interest

The authors declare no conflicts of interest.

## Data Availability

The data that support the findings of this study are openly available in The impact of living with inducible laryngeal obstruction at https://doi.org/10.5281/zenodo.14501352.
